# Supplementary figures and images for: Changes of diazotrophic communities in response to cropping systems in a Mollisol of Northeast China
Source: PeerJ. 2020 Jul 15;8:e9550. doi: 10.7717/peerj.9550 (PMC7368428; doi:10.7717/peerj.9550)

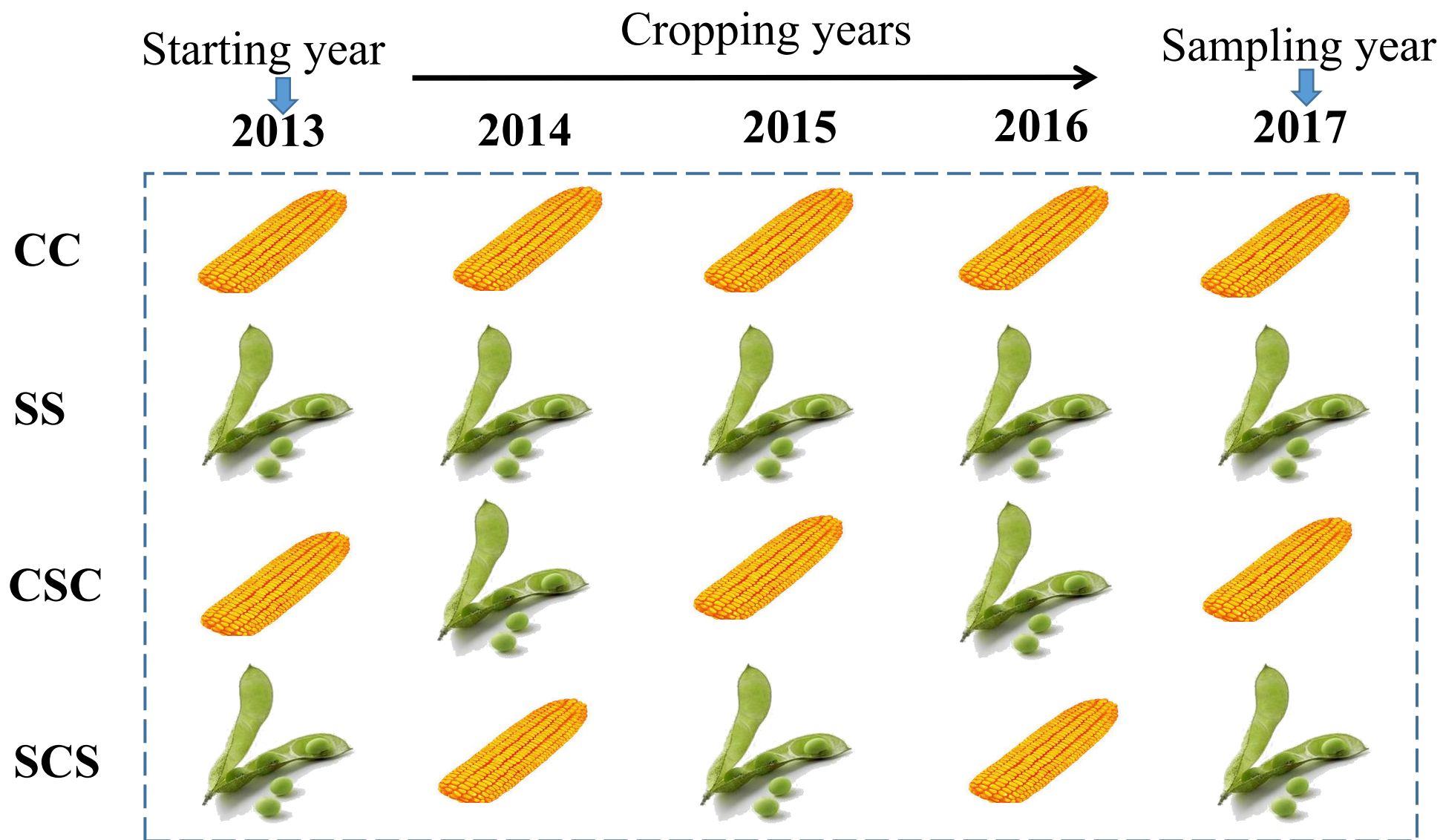

Supplement: Supplemental Information 2 — CC and SS represent the treatments of continuous cropping of corn and soybean, respectively; CSC and SCS represent the treatments of soybean-corn rotation for growing corn and soybean, respectively. [file peerj-08-9550-s002.pdf]
