# Supplementary material for: Changes of diazotrophic communities in response to cropping systems in a Mollisol of Northeast China
Source: PeerJ. 2020 Jul 15;8:e9550. doi: 10.7717/peerj.9550 (PMC7368428; doi:10.7717/peerj.9550)
Supplement: Supplemental Information 3 [file peerj-08-9550-s003.docx]

**Table S1** Adonis analysis the differences in diazotrophic community structures between treatments.

| Between treatments | *R*2 | *P* |
| --- | --- | --- |
| CC *vs* SS | **0.841**^a^ | **0.024** |
| CC *vs* CSC | **0.753** | **0.030** |
| CC *vs* SCS | **0.853** | **0.036** |
| SS *vs* CSC | **0.472** | **0.028** |
| SS *vs* SCS | 0.361 | 0.055 |
| CSC *vs* SCS | **0.661** | **0.035** |

^a^ Values in bold indicate significant correlation (*P* < 0.05).
