# Supplementary material for: Changes of diazotrophic communities in response to cropping systems in a Mollisol of Northeast China
Source: PeerJ. 2020 Jul 15;8:e9550. doi: 10.7717/peerj.9550 (PMC7368428; doi:10.7717/peerj.9550)
Supplement: Supplemental Information 4 [file peerj-08-9550-s004.docx]

**Table S2** Envfit analysis of the correlations between diazotrophic communities and soil factors

|  | *R*^2^ | *P* |  |
| --- | --- | --- | --- |
| pH | 0.065 | 0.661 |  |
| Total N | 0.151 | 0.334 |  |
| Total C | 0.288 | 0.100 |  |
| C/N | 0.291 | 0.101 |  |
| Total P | 0.038 | 0.784 |  |
| NH_4_^+^-N | 0.039 | 0.767 |  |
| NO_3_^-^-N | **0.378^a^** | **0.046** |  |
| Available P | **0.483** | **0.017** |  |
| Available K | **0.517** | **0.008** |  |
| Total K | 0.272 | 0.133 |  |

^a^ Values in bold indicate significant correlation (*P* < 0.05).
