# Supplementary material for: Changes of diazotrophic communities in response to cropping systems in a Mollisol of Northeast China
Source: PeerJ. 2020 Jul 15;8:e9550. doi: 10.7717/peerj.9550 (PMC7368428; doi:10.7717/peerj.9550)
Supplement: Supplemental Information 5 [file peerj-08-9550-s005.docx]

**Table S3** Mantel test to determine the correlations between diazotrophic communities and soil factors.

|  | *r* | *P* |
| --- | --- | --- |
| pH | 0.011 | 0.946 |
| Total N | -0.009 | 0.952 |
| Total C | 0.113 | 0.368 |
| C/N | 0.150 | 0.191 |
| Total P | -0.202 | 0.100 |
| Total K | 0.139 | 0.401 |
| NH_4_^+^-N | -0.234 | 0.111 |
| NO_3_^-^-N | 0.114 | 0.211 |
| Available P | **0.264**^a^ | **0.038** |
| Available K | 0.195 | 0.090 |

^a^ Values in bold indicate significant correlation (*P* < 0.05).
